# The Head–Toes–Knees–Shoulders Task as a Screening Tool for Kindergarten-Level Achievement

**DOI:** 10.3390/bs15111464

**Published:** 2025-10-28

**Authors:** Irem Korucu, Robert J. Duncan, Sabrina A. Kenny, Christopher R. Gonzales, Ahmad Ahmadi, Jasmine T. Karing, Megan M. McClelland

**Affiliations:** 1 Department of Psychology, Charles E. Schmidt College of Science, Florida Atlantic University, Boca Raton, FL 33431, USA; 2 Department of Human Development and Family Studies, College of Health and Human Sciences, Colorado State University, Fort Collins, CO 80523, USA; 3 Department of Human Development and Family Sciences, College of Health, Oregon State University, Corvallis, OR 97331, USA; 4 Morgridge College of Education, University of Denver, Denver, CO 80210, USA

**Keywords:** self-regulation, kindergarten achievement, screening tool, HTKS, HTKS-R

## Abstract

Prior research has consistently found significant associations between self-regulation and early academic achievement, yet the majority has focused primarily on understanding the magnitude of these linear associations rather than identifying the level at which self-regulation difficulties pose challenges for kindergarten achievement. In this longitudinal study, we examined the utility of a commonly used self-regulation task, the HTKS, and its updated version, the HTKS-R, as a potential screening tool for kindergarten-level achievement, using two different samples from the Pacific Northwest area of the United States. The probability of scoring at kindergarten-level for different scores on a self-regulation measure was examined along with the sensitivity, specificity, precision, and negative predictive value. Findings suggest that both the HTKS and the HTKS-R can be used as a screening tool, with the closest associations to mathematics, and when administered concurrently during the fall of kindergarten. Furthermore, both measures are best equipped to estimate who will be at/above kindergarten-level (precision), such that high performance on the HTKS is associated with a high likelihood of being at/above kindergarten-level in achievement. As a brief and easy-to-administer assessment, the HTKS tasks can provide insights for determining aspects of school readiness, including providing valuable information about children’s ability to perform on a self-regulation measure and children’s probability of performing at kindergarten-level on achievement.

## 1. Introduction

An important life transition for many children is moving from early childhood care (e.g., preschool or home environments) to kindergarten, a more structured learning environment. This complexly determined developmental process involves not only child-based variables, but also aspects of their interpersonal relationships with parents and teachers, and the impact of the settings in which their lived experiences occur. Within the more formal classroom environments of kindergarten, cognitive skills such as paying attention, following directions, transitioning between activities, and engaging in learning opportunities are important predictors of school success (McClelland & Cameron, 2012). Children’s executive function (EF), defined as a set of higher-order cognitive processes, includes inhibitory control, working memory, and cognitive flexibility (Blair, 2002; Diamond et al., 2002), and is thought to underlie self-regulated action (Bailey & Jones, 2019; Blair & Ku, 2022). Self-regulation, as a multidimensional construct that incorporates emotion, cognition, and behavior, is at the root of foundational skills needed for school success and has predicted school achievement from kindergarten through college completion (Blair & Diamond, 2008; McClelland et al., 2013; Robson et al., 2020; Willoughby et al., 2019).

Although research has found strong links between self-regulation and achievement (Blair & Razza, 2007; Fuhs et al., 2014), the point at which low levels of self-regulation become problematic for kindergarten-level achievement has not been explored. Addressing this gap is necessary, as recent legislation in the U.S. prioritized kindergarten entry assessments (KEA), and many states require KEAs (e.g., Arizona, Virginia, Delaware, Ohio, and Washington). These assessments aim to help educators better understand what children know and the level of skills and behaviors that they demonstrate upon kindergarten entry. States and practitioners have started using self-regulation and related skills (e.g., persistence, approaches to learning) as part of KEAs, making it important for researchers to provide meaningful interpretations that are easily understood by teachers, administrators, and practitioners. The current study explores how well a commonly used measure of self-regulation, the Head–Toes–Knees–Shoulders (HTKS) and its revised version (HTKS-R), works as a screening tool for kindergarten-level achievement (mathematics and literacy) during the fall of kindergarten. The study also investigates the differences associated with the HTKS as a screening tool when administered during the spring of prekindergarten and the fall of kindergarten and the implications of different cut-points.

### 1.1. Self-Regulation and Achievement

The preponderance of studies linking self-regulation to achievement suggests that these skills play a role in school success (e.g., Blair & Razza, 2007; Fuhs et al., 2014; Howard et al., 2022; Mann et al., 2017; McClelland et al., 2013; Robson et al., 2020). Researchers have consistently found evidence that the relationship between self-regulation and achievement persists above and beyond the effects of children’s IQ (Von Suchodoletz et al., 2013), socioeconomic status (McClelland & Wanless, 2012), and ethnicity (Wanless et al., 2011). Self-regulation skills are also considered important protective factors from many negative outcomes associated with socio-demographic risk factors (Luerssen & Ayduk, 2017; Obradović, 2010). Yet, there remains a gap in the literature on whether these measures could serve as useful screeners prior to or at school entry.

### 1.2. The Head–Toes–Knees–Shoulders

The HTKS is a commonly used self-regulation task that captures aspects of EF (inhibitory control, working memory, and cognitive flexibility) in overt behavior in early childhood (McClelland et al., 2014). The HTKS begins by assessing if children understand a paired opposite rule of touching their head (or shoulders) when asked to touch their toes (or knees) and vice versa. Over three sections of 17 practice items and 30 test items, the task builds in complexity by including additional opposite rules and eventually switching rules. The tasks require children to engage in core EF processes such as inhibitory control (suppressing a dominant response to touch their head when supposed to touch their toe), working memory (remembering changing rules from head-toes to knees-shoulders), and cognitive flexibility (flexibly switching rules as they change). It has been shown to be significantly correlated with other EF and self-regulation measures and is a strong indicator variable on latent EF factor models (Allan & Lonigan, 2011; Fuhs et al., 2014; McClelland et al., 2014; Schmitt et al., 2014). In the HTKS-R, a new downward extension was added to improve the variability of the scores and reduce floor effects (Gonzales et al., 2021). In the revised version, children are asked to verbally respond to instructions and say the opposite of what is asked (McClelland et al., 2021). The HTKS-R includes 37 test items and 22 practice items across four parts.

Previous studies have documented the strong psychometric characteristics of the HTKS and HTKS-R. Each version of the task typically has one of the strongest relations with achievement measures among other self-regulation measures (McClelland et al., 2014, 2021) and is valid and reliable in early childhood (Kenny et al., 2023). Scores on the tasks are also associated concurrently and longitudinally with academic achievement, as well as other measures of EF among diverse samples of children (Gonzales et al., 2021; McClelland & Cameron, 2012; McClelland et al., 2014; Wanless et al., 2011).

In addition to their strong psychometric characteristics, the HTKS tasks require no special equipment, are not linguistically demanding, are fun for young children, and are easy to administer over a short period of time (e.g., 5–10 min). It is also widely used for examining self-regulation in diverse racial, cultural, and socioeconomic groups (Ahmadi et al., 2025; Gestsdottir et al., 2014; Hee et al., 2018; Hernández et al., 2018; Lonigan et al., 2017; Zhang & Rao, 2017; Wanless et al., 2011). These characteristics have made the HTKS one of the most commonly used measures of self-regulation in the literature, and the HTKS has been identified as a strong candidate for screening prekindergarten children for school readiness (Lipsey et al., 2017). To the best of our knowledge, however, no studies have thus far incorporated the HTKS and HTKS-R tasks as screening tools to evaluate school readiness, particularly for kindergarten-level achievement. Consequently, the sensitivity, specificity, precision, and negative predictive value of the tasks in evaluating kindergarten-level achievement as a screening tool are unknown.

### 1.3. Importance of Screening Tools for Kindergarten Achievement

Understanding kindergarten-level achievement is an important issue, and using a measure of self-regulation is one possible way to evaluate academic achievement. Furthermore, self-regulation and achievement skills are simply one component of understanding how well children will be able to perform in school environments (i.e., school readiness). School readiness typically involves more than the individual child, consisting of the interactions between families, early childhood environments, schools, and the community. It is through these interactions that school readiness can be strengthened and promoted for all children. Unfortunately, many children enter school behind in foundational learning-related abilities (i.e., self-regulation and EF) and school readiness (i.e., understanding of numbers, letters, and words; Ghandour et al., 2021) that limit their ability to prosper in the increasingly demanding kindergarten environments. Understanding children’s preparedness for school is critical for identifying and providing services to those who are in need. Children’s performance on self-regulation and kindergarten-level achievement can inform how well children will be able to perform in school environments (i.e., school readiness). Gaps in the literature remain in determining the exact point at which early self-regulation skills significantly affect academic performance. Determination of a cut-score can help clarify the point at which low self-regulation may be problematic and early intervention may be needed. Furthermore, a cut score would provide a more precise indicator of when children are at risk for low academic performance, thus providing a starting point for early intervention.

### 1.4. Cut Score Analyses

Cut score analyses optimize sensitivity and specificity and correctly identify individuals who are below or above a certain threshold. Although this method is common in medical fields (i.e., identifying diabetes), it can be applied to many fields, such as education. By using cut-score analyses, we can identify whether a child has necessary skills to be successful in school. Although what is meant by “successful” can carry many meanings, one aspect of success in early childhood includes whether a child performs at kindergarten level in achievement. This approach establishes a practical tool that can be used and interpreted by novel assessors and convey an impactful insight as opposed to linear trends, which may be difficult to interpret.

It is also important to note that the objectives of a kindergarten achievement instrument may be different than those in the medical field (e.g., diabetes). Objectives for maximizing sensitivity and specificity should always consider the outcome of interest when determining if it is more important for an instrument to be more sensitive or specific. Given that maximizing sensitivity and specificity are conflicting objectives, we examined diagnostic information from a range of potential cut scores with the HTKS and HTKS-R.

Cut score and receiver operator characteristic analyses. In order to be a useful screening tool, the measure must have a cut-score that maximizes sensitivity and specificity at the same time, which are competing rather than complementary goals. If a very high cut-score is chosen, then sensitivity is maximized because virtually every child who scores above the extremely high cut-score will be at kindergarten-level in achievement. However, selecting an extremely high cut-score will also miss a number of children who are at kindergarten-level in achievement. One useful way to determine the cut score is by examining the receiver operator characteristic (ROC) curve. The area under the curve (AUC) is evaluated as an indicator of the sensitivity and specificity of a measure: AUC 0.90 is highly accurate, between 0.70 and 0.90 is moderately accurate, between 0.50 and 0.70 is low accuracy, and below 0.50 is a chance result for a test with no discrimination value (Fischer et al., 2003).

Based on the ROC curve, the Youden (1950) method identifies the point (referred to as Youden index *J*) of a continuous measure that is the maximum vertical distance between the curve and the diagonal line (i.e., *J* = maximum (sensitivity + specificity − 1), see Matsha et al., 2013 for the figure). Therefore, this point represents the score of an instrument that is the greatest improvement of the diagonal reference line, which would be the result of a useless test. The cut-scores can then be used to calculate sensitivity, specificity, precision, and negative predictive value. They are each important in interpreting a diagnostic test and they are products of a 2 × 2 chi-square table (see Table 1 as a reference for the following section).

Sensitivity. The sensitivity of the measure is the probability that a child scores over the determined cut-score given that the child is in fact positive on the outcome. In our example, it would be the probability that a child scores above the cut-score on the HTKS given that they perform at kindergarten-level in an achievement domain. The equation for sensitivity is:

$$Sensitivity=\;\frac{Number\;of\;True\;Positives\;(a)}{Number\;of\;True\;Positive\;\left(a\right)+Number\;of\;False\;Negatives\;(c)}$$

Specificity. The specificity of measure is the probability that a child scores below the determined cut-score given that the child is in fact negative on the outcome. In our example, it would be the probability that a child scores below the cut-score on the HTKS given that they perform below kindergarten-level in an achievement domain. The equation for specificity is:

$$Specificity=\;\frac{Number\;of\;True\;Negatives\;(d)}{Number\;of\;True\;Negatives\;\left(d\right)+Number\;of\;False\;Positives\;(b)}$$

Precision. The precision of a measure is the probability that a child is positive for the outcomes given that the child scores above the determined cut-score. In our example, it would be the probability that a child will be at kindergarten-level in an achievement domain if they score above the cut-score on the HTKS. The equation for precision is:

$$Precision=\;\frac{Number\;of\;True\;Positives\;(a)}{Number\;of\;True\;Positive\;\left(a\right)+Number\;of\;False\;Positives\;(b)}$$

Negative predictive value (NPV). The NPV of a measure is the probability that a child is negative for the outcomes given that the child scores below the determined cut-score. In our example, it would be the probability that a child will be below kindergarten-level in an achievement domain if they score below the cut-score on the HTKS. The equation for negative predictive value is:

$$NPV=\;\frac{Number\;of\;True\;Negatives\;(d)}{Number\;of\;True\;Negatives\;\left(d\right)+Number\;of\;False\;Negatives\;(c)}$$

### 1.5. The Present Study

The current study seeks to understand how well the HTKS and HTKS-R would function as a potential screening tool for children’s performance in achievement, specifically mathematics and literacy, in the fall of kindergarten. A recent meta-analysis report encompassing 69 studies across various cultural contexts indicated positive associations between the HTKS task and children’s performance in mathematics and literacy (Kenny et al., 2023). Furthermore, there is evidence suggesting that the HTKS is predictive of achievement in kindergarten and growth throughout the year (McClelland et al., 2007), yet the degree to which it could be used as a screening tool to provide information regarding achievement levels at school entry is unclear. More specifically, by determining the probability of scoring at kindergarten-level for different scores on the HTKS and HTKS-R, and the information gained from these tasks at different cut-scores, can provide additional information related to a broader concept of school readiness and potentially inform strategies to support the child’s school success.

The present study had two complementary aims, assessed at two different time periods, for determining how well the HTKS and HTKS-R perform as a screening tool for kindergarten-level mathematics and literacy. The first aim was to examine the predicted probability of scoring at kindergarten-level in achievement for different scores on the HTKS and HTKS-R. The second aim was to conduct cut-score analyses (sensitivity, specificity, precision, NPV) for different scores of the HTKS and HTKS-R tasks for kindergarten-level achievement. We examined the HTKS and HTKS-R tasks at two time points, in the spring of prekindergarten (roughly 6 months prior to kindergarten entry) and in the fall of the kindergarten year. Thus, when using the HTKS and HTKS-R scores during the prekindergarten year, the longitudinal association was assessed with roughly a 6-month lag. When using the HTKS and HTKS-R scores during the fall of kindergarten, a concurrent association was assessed.

## 2. Materials and Methods

### 2.1. Participants

The study includes data from two samples of children and families who participated in studies evaluating the HTKS measure: the Head–Toes–Knees–Shoulders task (HTKS) and its most current version, the Head–Toes–Knees–Shoulders task, revised edition (HTKS-R). In the HTKS sample, 424 children (49% female) and their families were included, recruited from 38 classrooms across 17 preschools located in rural areas in the Pacific Northwest of the United States. Fifty-five percent of the children were enrolled in Head Start preschools. Children attending Head Start met the poverty guidelines for Head Start attendance, indicating a low-income status for their families. The average age of the children at the study’s commencement was approximately 60 months old (*M_age_* = 59.8 in months, SD = 3.81). A parental demographic questionnaire reported the racial and ethnic composition as follows: White (63%), followed by Latino/Hispanic (19%), multiracial (13%), Asian/Pacific Islander (3%), and other ethnicities (2%). Parental education, self-reported, ranged from 0 to 30 years, with an average of approximately two years of college education (*M* = 14.40, *SD* = 3.68).

The HTKS-R sample included children and families qualifying for Head Start (*n* = 318, 53% female) recruited from 64 classrooms in 18 Head Start preschools in the Pacific Northwest of the United States. The children were about 64 months old on average (*M_age_* = 64.39 in months, *SD* = 3.66). Parents reported multiple racial/ethnic identities: 76% identified as “White,” 20% as “Latino/Hispanic,” and 4% as “other” in terms of race/ethnicity. According to the parent demographic questionnaire, the average education level of the primary caregiver was 12.20 years (*SD* = 2.66), with 67% reporting a high school education or less. Data and study materials are not publicly available by [blinded] Institutional Review Board (#4766), and this study was not preregistered.

### 2.2. Procedures

Data for both samples were collected at four different time points over the preschool and kindergarten years. The current study utilizes data from both studies collected at two time points, during the spring of prekindergarten and the fall of kindergarten, between 2011–2014 for the HTKS sample and 2016–2018 for the HTKS-R sample. In both of these time points, the HTKS and the achievement assessments were conducted concurrently. After obtaining parental consent and child assent, trained research assistants conducted the assessments with children in their schools over several 15–20-min sessions. In cases where caregivers or teachers reported a child’s proficiency in a language other than English, a bilingual assessor administered two subtests of the pre-language assessment screener (preLAS; S. E. Duncan & De Avila, 1998). For children whose home language was Spanish and scored 15 or more on the preLAS, all assessments were conducted in English; children scoring less than 15 points were assessed in Spanish (Rainelli et al., 2017). Children speaking a language other than Spanish and not passing the preLAS were not assessed at that time point. Spanish-speaking research assistants administered the preLAS at each point of the study, and children who had undergone the preLAS were assessed by bilingual assessors at each point.

### 2.3. Measures

HTKS and HTKS-R. For the HTKS, children are asked to do the opposite of what they were told (e.g., if told to touch their head, the child should touch their toes) across three sections, each including practice items followed by 10 test items. The task increases in complexity, requiring children to remember opposing rules involving four body parts (head, toes, knees, and shoulders). Children received 2 points for a correct response, 1 point for a self-corrected response, and 0 points for an incorrect response. Children only continued to the subsequent section if they received four or more points in the prior section of the task. Scores ranged from 0 to 94. Previous research with the HTKS has produced strong reliability and validity statistics within diverse samples (McClelland et al., 2014). The HTKS demonstrated high internal consistency in the current sample (Cronbach’s α = 0.93).

In the revised version of the HTKS, the HTKS-R, a new section was included at the start of the task, including practice items and 7 test items. In this section, children are asked to verbally respond to prompts, e.g., “When I say toes, you say head.” The rest of the task is the same as the HTKS. Children received 2 points for a correct response, 1 point for a self-corrected response, and 0 points for an incorrect response. Scores ranged from 0 to 118. Previous research with the HTKS-R has demonstrated evidence of strong reliability and validity (McClelland et al., 2021; Gonzales et al., 2021).

Academic Achievement. In both datasets, academic achievement was measured using subtests of the Woodcock Johnson-III (WJ-III, Woodcock et al., 2001). The Letter-Word Identification subtest of the Woodcock-Johnson Psycho-Educational Battery-III Tests of Achievement (WJ-III; Woodcock et al., 2001) or The Batería III Woodcock-Muñoz (Muñoz-Sandoval et al., 2005) measured emergent literacy, assessing letter skills and developing word-decoding abilities. The Applied Problem subtest of the Woodcock-Johnson Psycho-Educational Battery-III Tests of Achievement (WJ-III; Woodcock et al., 2001) or The Batería III Woodcock-Muñoz (Muñoz-Sandoval et al., 2005) measured early knowledge of mathematical operations, including counting, addition, and subtraction through practical problems. Total sum scores were utilized to obtain the grade equivalency score using the WJ-III software (Stata 18.0). Thus, in both samples, all academic variables are dichotomous measures of whether a child is performing at grade level in the fall of the kindergarten year.

### 2.4. Analytic Strategy

All analyses were conducted using Stata 18.0 (StataCorp. (College Station, TX, USA), 2023). The association between the HTKS and HTKS-R scores and the probability of performing at kindergarten-level in achievement was assessed using logistic regression models. First, the longitudinal association was assessed with the HTKS measures administered in the spring of prekindergarten and achievement administered in the fall of kindergarten. Second, the concurrent association was assessed with both measures in the fall of kindergarten. The primary focus was to evaluate how well a child’s HTKS scores could predict the probability of performing at kindergarten-level in achievement. Since we were interested in the unadjusted screening potential of the HTKS and HTKS-R, their capacity to identify children based on their performance on these tasks no covariates were included in the models. However, interactions were tested for each model to ensure that effects did not vary due to age, gender, or Spanish-speaking status. No significant interactions emerged, suggesting associations between the HTKS measures and achievement did not vary depending on children’s age, gender, or Spanish-speaking status. Second, the *roctab* command in Stata 18.0 was used to determine the model sensitivity, specificity, AUC, and ROC curve. Precision and NPV were calculated using the equations presented in the introduction. Results are presented for selected cut scores on the HTKS and HTKS-R. Furthermore, the Youden Index *J* (Youden, 1950) was used to determine the point in the HTKS assessments where sensitivity and specificity are maximized for a given outcome, with the sensitivity, specificity, precision, and NPV presented at this value. Although sensitivity and precision are often given prominence in screening tools, specificity and NPV are also considered in our analyses. Finally, missing data ranged from 0 to approximately 30% across study variables (see Table 2), and analyses were conducted using listwise deletion.

## 3. Results

### 3.1. HTKS

Descriptive Statistics. Descriptive statistics can be found in Table 2. In the spring of prekindergarten, the HTKS had an average score of 44.03 (*SD* = 29.03, range = 0–93). In the fall of kindergarten, children performed roughly 12 points better, with an average score of 56.65 (*SD* = 26.84, range = 0–94). In the fall of kindergarten, 81% of the sample performed at kindergarten-level in mathematics (249 of 309) and 73% at kindergarten-level in literacy (227 of 309).

Predicted Probability of Kindergarten-Level Achievement. All models showed significant effects for the HTKS measured in the spring of prekindergarten predicting kindergarten level achievement, with the closest association to mathematics (*b* = 0.056, *p* < 0.001, Odds Ratio (*OR*) = 1.06, CI [1.04, 1.07]), followed by literacy (*b* = 0.021, *p* < 0.001, *OR* = 1.02, CI [1.01, 1.03]). Across both achievement outcomes, scoring a “0” on the HTKS was associated with roughly a 0.46 probability of performing at kindergarten-level in achievement. For children scoring a “15” (1 *SD* below the mean), the probability increased to 0.60 for mathematics and 0.61 for literacy. For children scoring a “73” (1 *SD* above the mean), the probability increased to 0.97 for mathematics and 0.84 for literacy.

**Figure 1:**
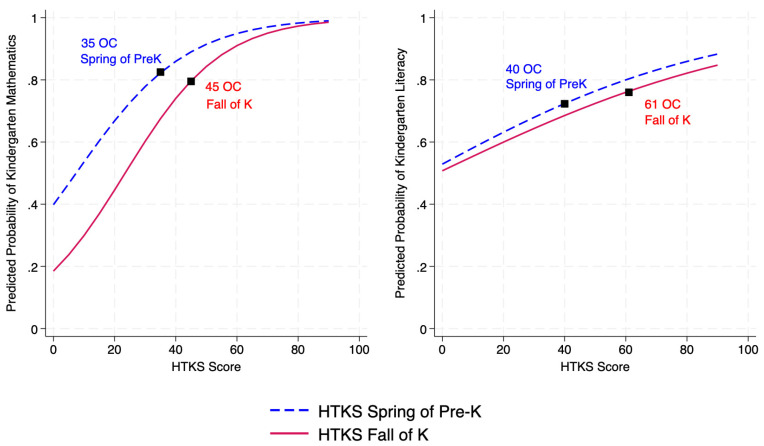
Predicted probabilities that children scored at kindergarten-level in mathematics and literacy based on their HTKS scores in the spring of preK and the fall of K. *Note.* ^OC^ is the optimal cut-score using Youden index.

When HTKS was assessed during the fall of kindergarten (i.e., concurrent with achievement assessment), both effects were significant for the HTKS predicting achievement with the closest association to mathematics (*b* = 0.06, *p* < 0.001, *OR* = 1.07, CI [1.05, 1.08]), and then literacy (*b* = 0.02, *p* < 0.001, *OR* = 1.02, CI [1.01, 1.03]). Scoring a “0” on the HTKS was associated with a 0.18 probability of performing at kindergarten-level in mathematics and a 0.51 probability in literacy. For children scoring a “30” (1 *SD* below the mean), the probability increased to 0.60 for mathematics and 0.64 for literacy. And for children scoring a “84” (1 *SD* above the mean, the probability increases to 0.97 for mathematics and 0.83 for literacy. Figure 1 presents the predicted probabilities that children scored at kindergarten level in mathematics and literacy based on their HTKS score during the spring of prekindergarten and the fall of kindergarten.

**Figure 2:**
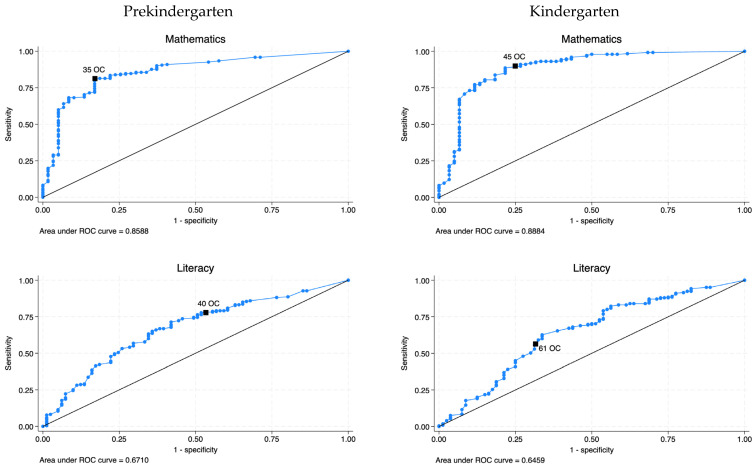
ROC curve analyses for academic achievement in the spring of pre-K and the fall of K for HTKS. *Note.* ^OC^ is the optimal cut-score using Youden index.

Cut-Score Information for the HTKS Predicting Kindergarten-Level Achievement. Consistent with the logistic regression results, cut-score analyses for the spring of prekindergarten showed that the largest AUC was observed for mathematics (0.82), and then literacy (0.65). Based on Fischer et al. (2003), these AUC reflect moderate accuracy (0.70–0.90) for predicting kindergarten-level mathematics and low accuracy (0.50–0.70) for kindergarten-level literacy. For math, a score of “35” on the HTKS in the spring of prekindergarten was selected as the optimal cut-score according to the Youden Index *J*, with sensitivity of 0.81 and specificity of 0.83 (see Figure 2). Importantly, for children who score a “35” or better, the probability of them later performing at kindergarten level in mathematics is 0.95 (i.e., the precision). For literacy, a score of “40” on the HTKS was selected, with sensitivity of 0.71 and specificity of 0.58.

Similarly, the cut-score analysis for the fall of kindergarten showed that the largest AUC was observed for mathematics (0.83), followed by literacy (0.64). The AUC reflected moderate accuracy for predicting kindergarten-level mathematics and low accuracy for predicting kindergarten-level literacy (Fischer et al., 2003). For mathematics, a score of “45” on the HTKS in the fall of kindergarten was selected as the optimal cut-score, with sensitivity of 0.89 and specificity of 0.78. Moreover, for children that score a “45” or better, the probability of them also being at kindergarten-level in mathematics is 0.82. A score of “61” was selected for literacy, with sensitivity of 0.63 and specificity of 0.66. The diagnostic information for the spring of prekindergarten and the fall of kindergarten HTKS score predicting kindergarten-level achievement is presented in Table 3.

### 3.2. HTKS-R

Descriptive Statistics. Descriptive statistics can be found in Table 2. In the spring of prekindergarten, the HTKS-R had an average score of 53.51 (*SD* = 30.23, range = 0–111). In the fall of kindergarten, children performed roughly 18 points better, with an average score of 71.96 (*SD* = 26.84, range = 0–118). In the fall of kindergarten, 62% of the sample performed at kindergarten level in mathematics (153 of 245) and 58% at kindergarten level in literacy (142 of 245).

Predicted Probability of Kindergarten-Level Achievement. Similar to HTKS results, all models showed significant effects for the HTKS-R measured in the spring of prekindergarten predicting kindergarten level achievement, with the closest association to mathematics (*b* = 0.035, *p* < 0.001, Odds Ratio (*OR*) = 1.04, CI [1.02, 1.05]), followed by literacy (*b* = 0.017, *p* < 0.05, *OR* = 1.02, CI [1.00, 1.03]). Across both achievement outcomes, scoring a “0” on the HTKS-R was associated with roughly a 0.26 probability of performing at kindergarten level in achievement. For children scoring a “23” (1 *SD* below the mean), the probability increased to 0.48 for mathematics and 0.29 for literacy. And for children scoring a “84” (1 *SD* above the mean), the probability increased to 0.89 for mathematics and 0.54 for literacy.

**Figure 3:**
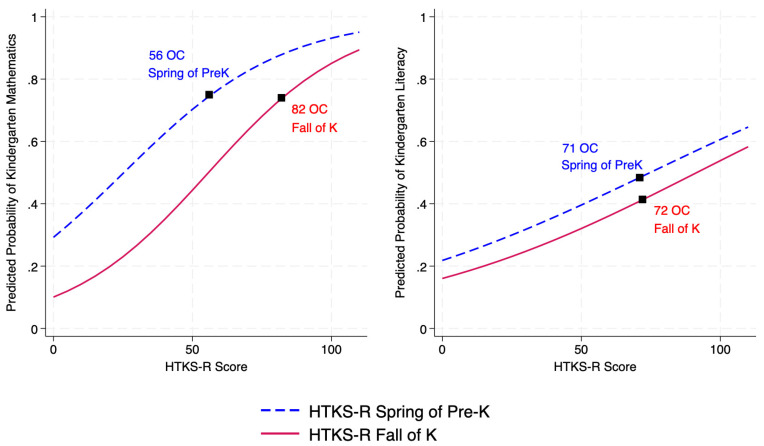
Predicted probabilities that children scored at kindergarten-level in mathematics and literacy based on their HTKS-R scores in the spring of preK and the fall of K. *Note.* ^OC^ is the optimal cut-score using Youden index. ROC = Receiver operating characteristic.

When HTKS-R was measured during the fall of kindergarten (i.e., concurrent with achievement assessment), all effects were also significant for the HTKS-R predicting achievement with the closest association to mathematics (*b* = 0.04, *p* < 0.001, *OR* = 1.04, CI [1.03, 1.05]), followed by literacy (*b* = 0.02, *p* < 0.001, *OR* = 1.02, CI [1.01, 1.03]). Scoring a “0” on the HTKS-R was associated with a 0.13 probability of performing at kindergarten level in achievement. For children scoring a “45” (1 *SD* below the mean), the probability increased to 0.40 and 0.30 for literacy. And for children scoring a “99” (1 *SD* above the mean), the probability increased to 0.85 for mathematics and 0.53 for literacy. The predicted probabilities that children scored at kindergarten-level in mathematics and literacy based on their HTKS-R score during the spring of prekindergarten and the fall of kindergarten are presented in Figure 3.

**Figure 4:**
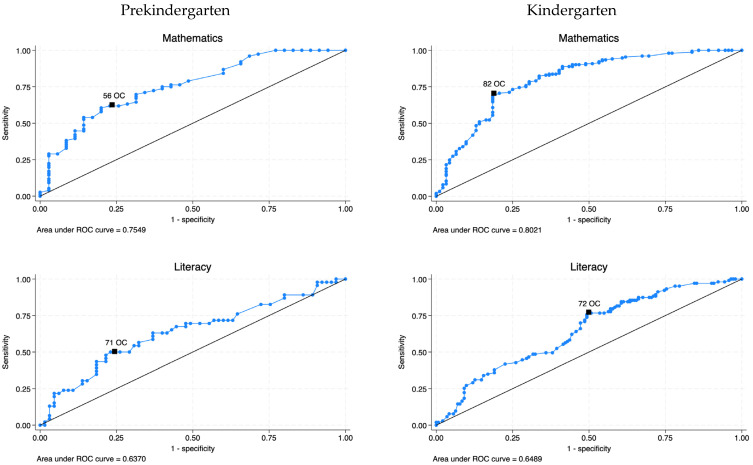
ROC curve analyses for academic achievement in the spring of pre-K and the fall of K for HTKS-R. *Note.* ^OC^ is the optimal cut-score using Youden index. ROC = Receiver operating characteristic.

Cut-Score Information for the HTKS-R in Predicting Kindergarten-Level Achievement. The cut-score analysis for the spring of prekindergarten HTKS-R score predicting kindergarten-level provided that the largest AUC was observed for mathematics (0.70) and then literacy (0.63). Based on Fischer et al. (2003), these AUC reflect moderate accuracy for predicting kindergarten-level mathematics and low accuracy for literacy. For mathematics, a score of “56” on the HTKS-R in the spring of prekindergarten was selected as the optimal cut-score according to the Youden Index *J*, with sensitivity of 0.61 and specificity of 0.80 (see Figure 4). For literacy, a score of “71” on the HTKS-R was selected, with sensitivity of 0.50 and specificity of 0.77.

The cut-score analysis for the fall of kindergarten HTKS-R score predicting kindergarten-level achievement also showed that the largest AUC was for mathematics (0.75), followed by literacy (0.63). The AUC reflected moderate accuracy for predicting kindergarten-level mathematics and low accuracy for predicting kindergarten-level literacy (Fischer et al., 2003). For mathematics, a score of “82” on the HTKS-R in the fall of kindergarten was selected as the optimal cut-score, with sensitivity of 0.69 and specificity of 0.82. A score of “72” was selected for literacy, with sensitivity of 0.76 and specificity of 0.51. The diagnostic information for the spring of prekindergarten and the fall of kindergarten HTKS-R score predicting kindergarten-level achievement is presented in Table 4.

In sum, a score of “35” on the HTKS was selected as the optimal cut-score for mathematics and “40” for literacy in the spring of prekindergarten. In the fall of kindergarten, a score of “45” for mathematics and a score of “61” for literacy on the HTKS was identified as the optimal cut-scores. For HTKS-R, a score of “56” was selected for mathematics and “71” for literacy in the spring of prekindergarten. In the fall of kindergarten, a score of “82” was identified for mathematics and “72” for literacy.

## 4. Discussion

This study explored a self-regulation measure, HTKS and HTKS-R, as a potential screening tool for children’s ability to perform at kindergarten-level in mathematics and literacy during the fall of kindergarten and longitudinally during the spring of prekindergarten. Prior literature has documented the strong links between EF, mathematics, and literacy (Ahmed et al., 2019; Schmitt et al., 2017). Our study supported these links and also extended prior research in unique ways by indicating that a commonly used self-regulation measure, HTKS and its revised version, the HTKS-R, can be used as a screening tool for kindergarten-level achievement in mathematics and literacy. Specifically, the closest associations were observed with mathematics compared to literacy and when the HTKS is administered concurrently during the fall of kindergarten compared to during the spring of pre-kindergarten. Further, getting a high score on the HTKS and HTKS-R is associated with large increases in the probability of being at kindergarten level in achievement in mathematics and literacy. The HTKS also demonstrated precision and sensitivity in predicting kindergarten achievement outcomes.

Extending the prior research examining the linear trends of the HTKS and academic outcomes, the dichotomizing of the academic achievement variables in the present study allowed us to determine whether the two versions of the HTKS could predict children’s achievement of grade-level performance in the fall of kindergarten. Analyzing the predicted probabilities revealed that all models were statistically significant, with associations consistently favoring mathematics over literacy. These findings suggest that children scoring higher on the HTKS (and HTKS-R) are more likely to achieve grade-level kindergarten competencies both concurrently and longitudinally (i.e., HTKS scores in preschool predicting grade-level achievement six months later in kindergarten). Conversely, a more concerning interpretation is that lower self-regulation skills during the preschool and kindergarten years may put children at greater risk of not meeting grade-level standards.

Upon investigating the potential of the HTKS and HTKS-R to predict children’s likelihood of achieving kindergarten-level competencies in mathematics and literacy, we also evaluated these measures’ screening potential, both longitudinally and concurrently. Our findings indicate that the HTKS and HTKS-R are moderately accurate in predicting grade-level mathematics performance but show somewhat lower accuracy for predicting grade-level literacy performance. These outcomes align with our logistic regression analyses and prior research, highlighting self-regulation as a more robust predictor of success in mathematics than literacy (Clements et al., 2016; Cragg & Gilmore, 2014; G. J. Duncan et al., 2007; Schmitt et al., 2017). This difference may be attributed to literacy development being less directly dependent on EF and self-regulation skills than mathematics. For instance, early literacy success is heavily influenced by environmental factors, such as language exposure, access to books and other literacy-enriching materials, and caregiver-child interactions (Tamis-LeMonda et al., 2019; Schmitt et al., 2011). These supports can serve as protective mechanisms for children, even when their EF skills are not well-developed (Korucu et al., 2020). In contrast, lagging EF and self-regulation skills may place children at higher risk for mathematics-related challenges, as children often do not have comparable experiences to promote mathematics development (Purpura et al., 2017).

The HTKS and HTKS-R are frequently commended for their strong psychometric properties and ease of administration, scoring, and interpretation procedure (Lipsey et al., 2017). Given these advantages and their widespread international application in research (e.g., Kenny et al., 2023), this study significantly contributes to the self-regulation measurement literature. We propose that the findings hold three crucial implications for future research, practice, and policy: (a) they offer insights into the predictive capabilities of a widely-used self-regulation measure that could serve as a potential starting point for educational decision-making (b) they elucidate some of the complexities of the self-regulation and academic relationship for a broader audience, and (c) they bolster research and advocacy efforts calling for practices and policies that support children with more nascent self-regulation skills in educational settings.

### 4.1. Supporting Education Decision-Making

Numerous studies have indicated that self-regulation skills are a consistent predictor of children’s academic success (e.g., Gestsdottir et al., 2014; Hee et al., 2018; Kenny et al., 2023; Lipsey et al., 2017; McClelland et al., 2014, 2021; Schmitt et al., 2014, 2017). A consistent link documented between a child’s self-regulation capacity and academic achievement, prompted both researchers and educators to advocate for more strategies and interventions to enhance children’s self-regulation. However, it is essential to acknowledge that in research investigating the links between self-regulation and academic performance (including the studies mentioned above), both are measured as continuous variables. It is, therefore, challenging to make inferences beyond that higher scores on self-regulation measures tend to yield higher scores on academic performance measures. What remains uncertain from these studies is whether there is a point at which a child’s self-regulation level results in optimal (or nonoptimal) academic performance, such as meeting grade-level competencies.

Because it is unclear when (or at what point) lower or higher levels of self-regulation become an academic liability or asset, early childhood professionals may continue to face challenges in obtaining actionable insights into the relationship between self-regulation and academic performance. We propose that the cut scores suggested in the current study build upon traditional correlational insights about the relationship between self-regulation and academic performance. They can offer a more precise indicator of when self-regulation might significantly influence academic performance, providing a valuable starting point for educational decision-making. In other words, these defined thresholds could be one factor that assists researchers, educators, and practitioners in identifying those who might benefit the most from self-regulation interventions or deciding at what point self-regulation should become a targeted outcome. Therefore, integrating brief assessments of self-regulation into existing kindergarten entry assessments could provide educators with data to inform instructional planning and targeted supports.

### 4.2. Elucidating the Self-Regulation and Academic Achievement Association

Most researchers aim for their findings to positively impact the development of children. However, scientific evidence generally lives in the research realm and for the research findings to make an impact in the development of children, they need to be translated for the policy-makers (Shonkoff & Bales, 2011). Specifically, policymakers may struggle to fully understand the complexities of self-regulation and its academic implications, including its significance in policy development. This study reveals that a child’s self-regulation in preschool or in kindergarten could predict a child’s grade-level performance in kindergarten; by focusing on what policymakers can relate to (i.e., grade equivalency) as opposed to a linear trend, our findings may offer accessible and impactful information. In other words, this study suggests that self-regulation influences not just the degree of academic achievement but the likelihood that a child is performing at a certain grade level, providing a more convincing case for policies that support self-regulation development in early learning contexts (McClelland et al., 2017).

### 4.3. Bolstering Prior Research and Advocacy Efforts

Strong self-regulation skills are helpful for children’s school success, whereas lower levels of self-regulation may make it more difficult for children to meet academic and social demands (Robson et al., 2020). The present study substantiates these findings by providing evidence that this relationship remains stable despite using a method that dichotomizes academic performance. We encourage professionals and policymakers to support policies and practices that provide children with emerging self-regulation skills the same opportunities for academic success as their more regulated peers. For instance, schools can focus on implementing evidence-based curricula and strategies that support children’s acquisition of the self-regulation skills that have been consistently identified as foundational to school success, in addition to focusing on academic competencies (Durlak et al., 2011; McClelland et al., 2017). Previous research suggests that children’s self-regulation skills can be improved through targeted interventions, and especially those that are integrated into everyday practice (Diamond & Lee, 2011; McClelland et al., 2019; Schmitt et al., 2015). Professional development that equips educators with strategies to foster self-regulation skills within everyday instruction could therefore be a valuable avenue for enhancing students’ readiness. In addition, adjustments can be made to the school environment to better cater to the varying levels of self-regulation development in children, ensuring that those with less developed self-regulation skills do not fall behind academically. 

### 4.4. Limitations and Future Directions

This study has direct implications for children’s school readiness and suggests that the HTKS and its revised version, the HTKS-R, can be used as a screening tool for school readiness. However, a few limitations should be noted. First, although our samples include diverse participants, it is important to highlight that the majority of the participants in this study attended Head Start centers, which may represent the experiences of children from families with low income and may not be generalizable across different samples. Future research is needed to replicate these findings with different race, income, and family education level compositions. Relatedly, because this sample is not a nationally representative sample, and the HTKS scores are not standardized against a national norming sample, future research is needed to determine whether the same cut scores would emerge with different samples. Moreover, the diagnostic properties of both measures were moderately accurate in predicting kindergarten-level mathematics but showed lower accuracy for kindergarten-level literacy. Future research could further focus on improving measures’ diagnostic characteristics for literacy.

While our findings suggest that the HTKS and the HTKS-R can serve as promising screening tools for kindergarten-level achievement, it is important to caution against using a single measure at a single time point for educational decision-making. Screening should incorporate multiple sources of information (e.g., teacher observations, curriculum-based assessments) across multiple time points to avoid misclassification. Further, identifying children as at-risk or on-track based on a screening tool may directly or indirectly influence teachers’ expectations or instructional decisions that may have implications for children’s self-perception. Therefore, the HTKS tasks should be considered as one component of a broader monitoring of school readiness skills rather than a stand-alone screening tool. Relatedly, children’s early academic achievement is influenced by a dynamic interplay of multiple factors, ranging from broader contextual factors (e.g., instructional quality, home learning environment) to individual variables (e.g., motivation). Our findings should be interpreted within this broader ecological framework, recognizing self-regulation as one important contributor among other factors that influence children’s early academic achievement.

## 5. Conclusions

This study investigated how well a self-regulation measure, the HTKS and its revised version, the HTKS-R, predicted children’s ability to perform at kindergarten-level mathematics and literacy. By examining grade-level achievement scores and employing cut-score analysis (i.e., roc curve analysis), this work expands prior research that examined a linear effect between self-regulation and achievement and highlights that the HTKS measures can be used as a screening tool for kindergarten-level achievement. Considering the need for screening tools for school readiness and their potential impact on children’s development, our findings highlight the practical utility of using cut-scores on the HTKS for predicting school readiness. The results of this study can be used to identify and support children’s self-regulation and early academic achievement as they make the transition to formal schooling.

## Figures and Tables

**Table 1:** Possible Outcomes Associated with Diagnostic Tools.

	Fall Kindergarten Achievement Score	
Fall HTKS Score	At Kindergarten-Level	Not At Kindergarten-Level
HTKS (ready)	True Positive (a)	False Positive (b)
HTKS (not ready)	False Negative (c)	True Negative (d)

**Table 2:** Descriptive Statistics for HTKS (Sample 1) and HTKS-R (Sample 2) for the Spring of Prekindergarten and Fall of Kindergarten.

**HTKS**	**Prekindergarten**				**Kindergarten**			
	***N***	**Mean (SD)/%**	**Range**	**% Missing**	***N***	**Mean (SD)/%**	**Range**	**% Missing**
Age in months	408	61.53 (3.94)	41.52–70.37	6.2	322	67.78 (3.68)	58.20–78.04	26
Sex	435			0	435			0
Male		51.72%						
Female		48.28%						
Language (Spanish speaking status)	424	15.33%		2.5	435	14.94%		0
HTKS	393	44.04 (29.03)	0–93	9.7	306	56.65 (26.84)	0–94	29.7
Kindergarten level mathematics		-			309	80.58%		29.0
Kindergarten level literacy		-			309	73.49%		29.0
**HTKS-R**	**Prekindergarten**				**Kindergarten**			
	***N***	**Mean (SD)/%**	**Range**	**% Missing**	***N***	**Mean (SD)/%**	**Range**	**% Missing**
Age in months	266	61.45 (3.65)	54.36–68.32	16.1	246	67.34 (3.68)	59.61–73.87	22.4
Sex	313			1.3				
Male		51.74%						
Female		47.00%						
Language (Spanish speaking status)	278	10.00%		12.3	246	6.25%		22.4
HTKS-R	137	53.51 (30.23)	0–111	56.8	246	71.96 (32.21)	0–118	22.4
Kindergarten level mathematics		-			245	62.45%		22.7
Kindergarten level literacy		-			245	42.04%		22.7

**Table 3:** Diagnostic Information for the Spring of Prekindergarten and Fall of Kindergarten HTKS Predicting Kindergarten-level Achievement.

Prekindergarten					Kindergarten				
	**Mathematics (AUC = 0.82)**					**Mathematics (AUC = 0.83)**			
**HTKS Score**	**Sensitivity**	**Specificity**	**Precision**	**NPV**	**HTKS Score**	**Sensitivity**	**Specificity**	**Precision**	**NPV**
10	0.88	0.63	0.90	0.55	10	0.98	0.50	0.88	0.86
20	0.85	0.73	0.93	0.54	20	0.95	0.58	0.90	0.73
30	0.83	0.78	0.94	0.52	30	0.93	0.63	0.91	0.69
**35 ^OC^**	**0.81**	**0.81**	**0.95**	**0.51**	40	0.91	0.72	0.93	0.66
40	0.74	0.83	0.94	0.44	**45 ^OC^**	**0.89**	**0.75**	**0.94**	**0.63**
50	0.67	0.92	0.97	0.40	50	0.84	0.82	0.95	0.55
60	0.49	0.95	0.97	0.31	60	0.71	0.92	0.97	0.43
70	0.29	0.95	0.96	0.25	70	0.47	0.93	0.97	0.30
80	0.11	0.98	0.94	0.21	80	0.24	0.95	0.95	0.23
90	0.02	100	0.92	0.20	90	0.04	100	1.00	0.20
	**Literacy (AUC = 0.65)**					**Literacy (AUC = 0.64)**			
10	0.83	0.36	0.78	0.44	10	0.92	0.20	0.76	0.46
20	0.79	0.42	0.79	0.43	20	0.88	0.26	0.77	0.44
30	0.81	0.51	0.80	0.44	30	0.87	0.31	0.78	0.45
**40 ^OC^**	**0.78**	**0.48**	**0.82**	**0.43**	40	0.84	0.36	0.78	0.45
50	0.63	0.65	0.85	0.39	50	0.77	0.46	0.80	0.42
60	0.48	0.78	0.85	0.35	**61 ^OC^**	**0.63**	**0.66**	**0.83**	**0.39**
70	0.29	0.88	0.86	0.31	70	0.44	0.75	0.82	0.32
80	0.10	0.95	0.86	0.28	80	0.22	0.85	0.78	0.28
90	0.02	0.99	0.85	0.27	90	0.04	0.98	0.73	0.27

*Note*. AUC = area under the ROC curve. ^OC^ is the optimal cut-score using Youden index.

**Table 4:** Diagnostic Information for the Spring of Prekindergarten and Fall of Kindergarten HTKS-R Predicting Kindergarten-level Achievement.

Prekindergarten					Kindergarten				
	**Mathematics (AUC = 0.70)**					**Mathematics (AUC = 0.75)**			
**HTKS-R Score**	**Sensitivity**	**Specificity**	**Precision**	**NPV**	**HTKS-R Score**	**Sensitivity**	**Specificity**	**Precision**	**NPV**
10	0.100	0.00	0.64	1.0	10	0.100	0.05	0.64	1.0
20	0.97	0.29	0.65	0.83	20	0.100	0.11	0.65	1.0
30	0.76	0.54	0.65	0.51	30	0.95	0.39	0.72	0.82
40	0.68	0.69	0.65	0.50	40	0.90	0.51	0.75	0.76
50	0.64	0.69	0.65	0.47	50	0.89	0.59	0.78	0.76
**56 ^OC^**	**0.61**	**0.80**	**0.66**	**0.48**	60	0.84	0.61	0.78	0.69
60	0.54	0.83	0.65	0.45	70	0.79	0.67	0.80	0.66
70	0.45	0.86	0.64	0.42	80	0.71	0.77	0.83	0.61
80	0.36	0.91	0.63	0.40	**82 ^OC^**	**0.69**	**0.82**	**0.86**	**0.61**
90	0.21	0.97	0.62	0.36	90	0.52	0.84	0.84	0.51
100	0.05	0.97	0.58	0.32	100	0.33	0.92	0.88	0.45
110	0.00	1.0	0.57	0.32	110	0.14	0.97	0.88	0.40
	**Literacy (AUC = 0.63)**					**Literacy (AUC = 0.63)**			
10	0.98	0.06	0.43	0.80	10	0.100	0.03	0.43	1.0
20	0.89	0.11	0.42	0.58	20	0.98	0.21	0.43	0.80
30	0.72	0.37	0.43	0.65	30	0.92	0.25	0.47	0.82
40	0.70	0.52	0.45	0.71	40	0.86	0.34	0.49	0.77
50	0.67	0.55	0.45	0.71	50	0.84	0.39	0.50	0.77
60	0.57	0.68	0.46	0.69	60	0.80	0.42	0.59	0.74
70	0.50	0.75	0.46	0.68	70	0.77	0.49	0.52	0.74
**71 ^OC^**	**0.50**	**0.77**	**0.47**	**0.68**	**72 ^OC^**	**0.76**	**0.51**	**0.53**	**0.74**
80	0.39	0.82	0.46	0.65	80	0.64	0.54	0.50	0.68
90	0.24	0.91	0.45	0.63	90	0.49	0.68	0.53	0.65
100	0.07	0.97	0.43	0.59	100	0.34	0.85	0.61	0.64
110	0.00	1.0	0.43	0.59	110	0.15	0.93	0.60	0.60

*Note*. AUC = area under the ROC curve. ^OC^ is the optimal cut-score using Youden index.

## Data Availability

Data and study materials are not publicly available by Oregon State University Institutional Review Board (#4766).
